# Isotope Effects in the Association Reactions of Methyl and Ethyl Iodide Cations*

**DOI:** 10.6028/jres.081A.016

**Published:** 1977-04-01

**Authors:** L. Wayne Sieck

**Affiliations:** Institute for Materials Research, National Bureau of Standards, Washington, D. C. 20234

**Keywords:** Alkyl iodides, ion-molecule reactions, isotope effects, mass spectrometry, photo-ionization, rate constants

## Abstract

Rate coefficients for production of stabilized dimeric parent cations at 295 K have been determined in CH_3_I, CD_3_I, CH_3_I—CD_3_I mixtures, C_2_H_5_I, CH_3_CD_2_I, CD_3_CH_2_I, and C_2_D_5_I. These processes are the most rapid reported for association reactions, the various individual values falling within the limits 0.33 × 10^−24^ (CH_3_I) and 10.1 ×10^−24^ cm^6^ molecule^−2^ sec^−1^ (C_2_H_5_I). The temperature dependence of the stabilization coefficients in CH_3_I and CD_3_I was also measured over the range 220 ± 3 to 320 ± 1 K, as well as the efficiencies of other third bodies in the stabilization process. The differences observed for the variously labelled analogues are interpreted in terms of vibrational level (energy) depression upon deuteration, which affects the intrinsic lifetime of the collision complex.

## 1. Introduction

Several years ago [l]^1^ we reported a study of parent dimer cation formation in the methyl halides using high pressure photoionization mass spectrometry, which extended earlier work by Hamill and co-workers [2–4] concerned with association ion production in the ethyl and propyl bromides and iodides. The common characteristic of these systems is the high overall efficiency for the third order process

$$A^{+}+2A\to {\left(A\right)}_{2}^{+}+A$$ (1)

where A^+^ denotes the parent molecular ion. More recently [5] we completed an investigation of dimer formation in low molecular weight aromatic hydrocarbons in which we found that production of ${\left(C_{6}D_{6}\right)}_{2}^{+}$ in C_6_D_6_ was a factor of 2.7 times faster than ${\left(C_{6}H_{6}\right)}_{2}^{+}$ formation in C_6_H_6_ at 295 K. In view of this pronounced isotope effect, we initiated a new photoionization study involving the methyl and ethyl iodides emphasizing the effect of isotopic substitution on the rate coefficient for process 1. These molecules were considered to be ideal systems for detailing some of the general factors influencing stabilization of ion-molecule complexes since >90 percent of the parent ions produced initially via photoionization are in *v* = 0 of the ground electronic state [6] (internal energy effects are minimal), the variously labelled deuterium analogues are readily available, and the room temperature stabilization coefficients are sufficiently high to permit experiments at reduced collision frequencies under controlled conditions.

## 2. Experimental Procedure

The NBS high pressure photoionization mass spectrometer equipped with a field-free reaction chamber was used for all experiments. Ionization of methyl iodide (I.P. ^2^E_1/2_ = 9.54 eV) and ethyl iodide (I.P. ^2^E_1/2_ = 9.34 eV, I.P. ^2^E_3/2_ = 9.93 eV) [7] was induced via photoabsorption at 123.6 nm (10.0 eV) using a Kr resonance lamp equipped with MgF_2_ optics. Measurements other than those at 295 K were taken with a variable temperature modification described elsewhere [8].

## 3. Results

### 3.1. General Behavior

**Figure 1:**
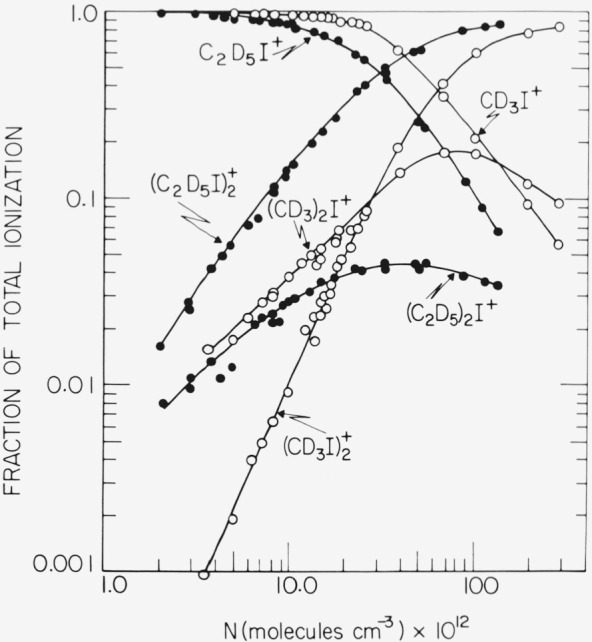
Composite spectra obtained in photoionized *CD*_3_*I* and *C*_2_*D*_5_*I* as a function of concentration.

Typical composite spectra found for methyl and ethyl iodides as a function of pressure are given in figure 1 for CD_3_I and C_2_D_5_I. In every system the competing reactions

$${\text{RI}}^{+}+\text{RI}\overset{k_{2}}{⇄}{\left(\text{RI}\right)}_{2}^{+*}$$ (2)

followed by

$${\left(\text{RI}\right)}_{2}^{+}\to R_{2}I^{+}+I$$ (3)

or

$${\left(\text{RI}\right)}_{2}^{+*}+\text{RI}\to {\left(\text{RI}\right)}_{2}^{+}+\text{RI}$$ (4)

where RI = methyl or ethyl iodide, were observed. Process 2 involves bimolecular formation of a transient collision complex ${\left(\text{RI}\right)}_{2}^{+*}$, which either dissociates to reform reactants or eliminates an iodine atom (process 3) at lower densities. At higher densities collisional stabilization (process 4) is facilitated, as indicated in figure 1 by the rapid formation of ${\left({\text{CD}}_{3}I\right)}_{2}^{+}$ and ${\left(C_{2}D_{5}I\right)}_{2}^{+}$ and the concurrent decrease in the relative fractional yields of the respective dialkyliodium ions.

### 3.2. Reaction Rates

Rate coefficients for process 3 at 295 K were derived from the initial slopes of the growth curves found for R_2_I^+^ when the respective fractional yields were plotted in semilogarithmic form versus RI concentration. Residence times (*τ*’s) for various RI^+^ ions at the zero pressure limit were approximated by first evaluating average *τ*’s for several ions of differing *m/e* ratios at 295 K under molecular flow conditions (<l–2 millitorr)^2^ using calibration reactions [9] and assuming the usual (mass)^1/2^ dependence for relative *τ*’s. Calibration reactions included $t—C_{4}H_{9}^{+}+{\text{CH}}_{3}{\text{NH}}_{2}\to {\text{CH}}_{3}{\text{NH}}_{3}^{+}\left(k=1.30\pm 0.15\times 10^{-9}\right)$, 3 $C_{3}H_{7}^{+}+i—C_{4}H_{10}\to C_{4}H_{9}^{+}\left(k=4.2+0.2\times 10^{-10}\right),C_{3}H_{6}^{+}+C_{3}H_{6}\to \text{products}\left(k=8.0\pm 0.5\times 10^{-10}\right)$, and ${\text{NH}}_{3}^{+}+{\text{NH}}_{3}\to {\text{NH}}_{4}^{+}\left(k=1.9\pm 0.2\times 10^{-9}\right)$. Third order stabilization coefficients (process 4) were calculated from the initial slopes of the logarithm^2^ of the fractional intensities of ${\left(\text{RI}\right)}_{2}^{+}$ versus (concentrations) using the same respective *τ*’s for the various RI^+^ ions. Residence times at other temperatures were calculated by applying the appropriate velocity corrections.

### 3.3. Methyl Iodides

Rate coefficients for production of dialkyliodium ions in CH_3_I, CD_3_I, and CH_3_—CD_3_I (1:1) mixtures at 295 K are given in table 1, along with values for CH_3_I and CD_3_I at 220 ± 3 and 320 ± 1 K. Within the indicated reproducibility limits, the coefficients for (CH_3_)_2_I^+^ formation in CH_3_I and (CD_3_)_2_I^+^ in CD_3_I were invariant at 2.4 ± 0.1 and 2.25 ± 0.1 × 10^−11^, respectively, over this temperature range. The overall 295 K coefficient for production of (CX_3_)_2_I^+^ in CH_3_I—CD_3_I (1:1) mixtures was found to be 2.3 ± 0.2 × 10^−11^. Products indicating isotopic scrambling such as (CH_2_D)(CH_3_)I^+^, (CD_2_H)(CH_3_)I^+^, etc., were not observed; the only second order products detected were (CH_3_)_2_I^+^, (CH_3_)(CD_3_)I^+^, and (CD_3_)_2_I^+^, with an essentially statistical distribution (0.26:0.50:0.24).

**Figure 2:**
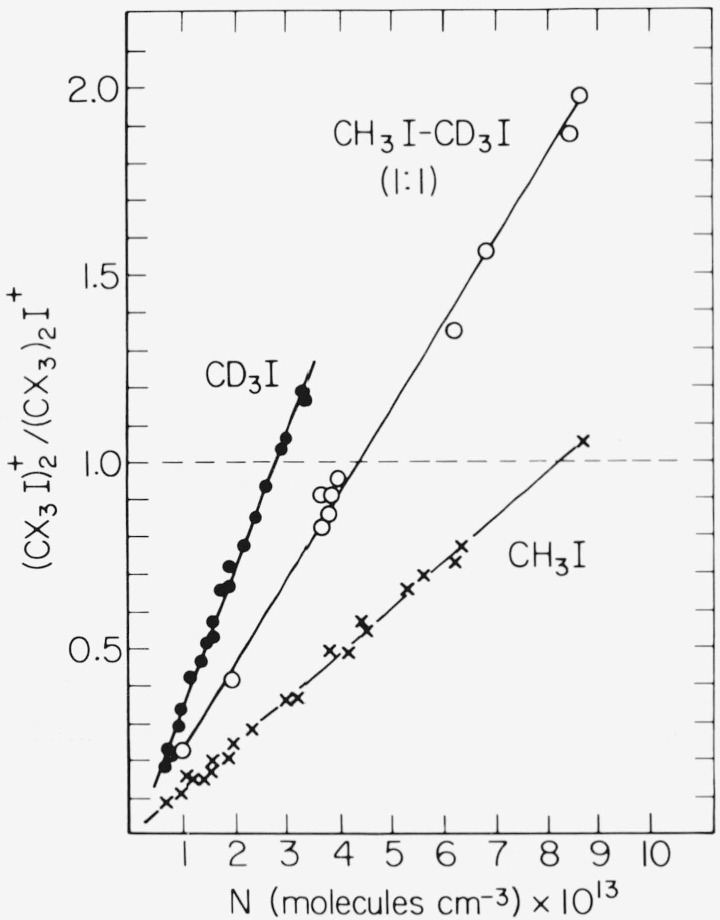
${\left({\text{CX}}_{3}I\right)}_{2}^{+}/{\left({\text{CX}}_{3}\right)}_{2}I^{+}$ ratio (*X* = *H* or *D*) versus concentration in *CH*_3_*I*, *CH*_3_*I*—*CD*_3_*I* mixtures (1:1), and *CD*_3_*I*.

**Figure 3:**
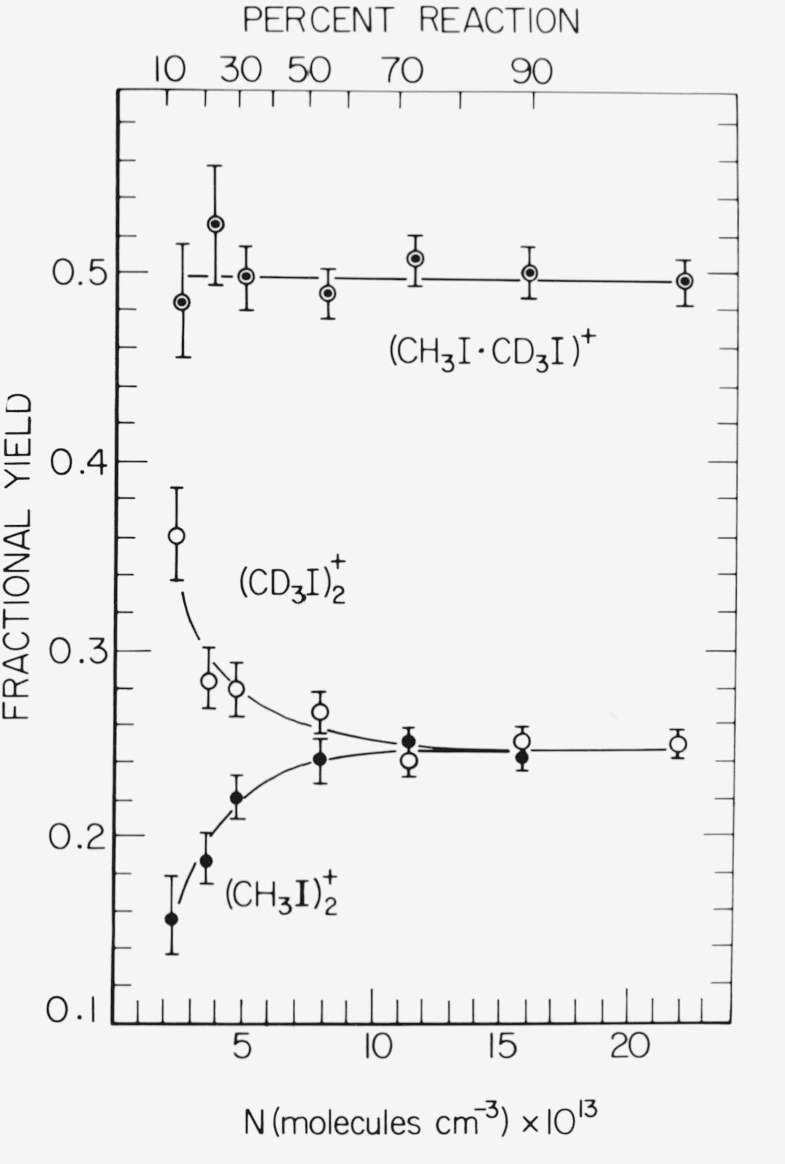
Fractional yields of ${\left({\text{CH}}_{3}I\right)}_{2}^{+},{\left({\text{CH}}_{3}{\text{I—CD}}_{3}I\right)}^{+}$, and ${\left({\text{CD}}_{3}I\right)}_{2}^{+}$ versus concentration in *CH*_3_*I*—*CD*_3_*I* mixtures (1:1).

In contrast to the near equivalency of the halogen elimination rates, substantial isotope effects were found in the third order coefficients for collisional stabilization of ${\left({\text{CX}}_{3}I\right)}_{2}^{+*}\left(X\;=\;\text{H or D}\right)$. Data for CH_3_I, CD_3_I, and CH_3_I—CD_3_I (1:1) mixtures are shown in figure 2, which gives the appropriate ${\left({\text{CX}}_{3}I\right)}_{2}^{+}/{\left({\text{CX}}_{3}\right)}_{2}I^{+}$ ratios versus CX_3_I concentration. Relative stabilization efficiencies are given by the slope ratios after correction for the slight differences found in the production rates for (CX_3_)_2_I^+^, and were determined to be 1.0, 1.8(3), and 2.8(0) in CH_3_I—CD_3_I (1:1), and CD_3_I respectively. Absolute values, as well as those determined at 220 ± 3 and 320 ± 1 K in CH_3_I and CD_3_I, are listed in table 1. The isotopic compositions of the ${\left({\text{CX}}_{3}I\right)}_{2}^{+}$ dimer ions formed in a (1:1) CH_3_I—CD_3_I mixture as a function of concentration and percent reaction of CX_3_I^+^ are given in figure 3. Again no evidence was found for hydrogen-scrambled species such as $\left({\text{CH}}_{2}D\right){\left({\text{CD}}_{3}\right)}_{2}^{+}$, etc.; i.e., the methyl groups retain their isotopic integrity during stabilization.

**Figure 4:**
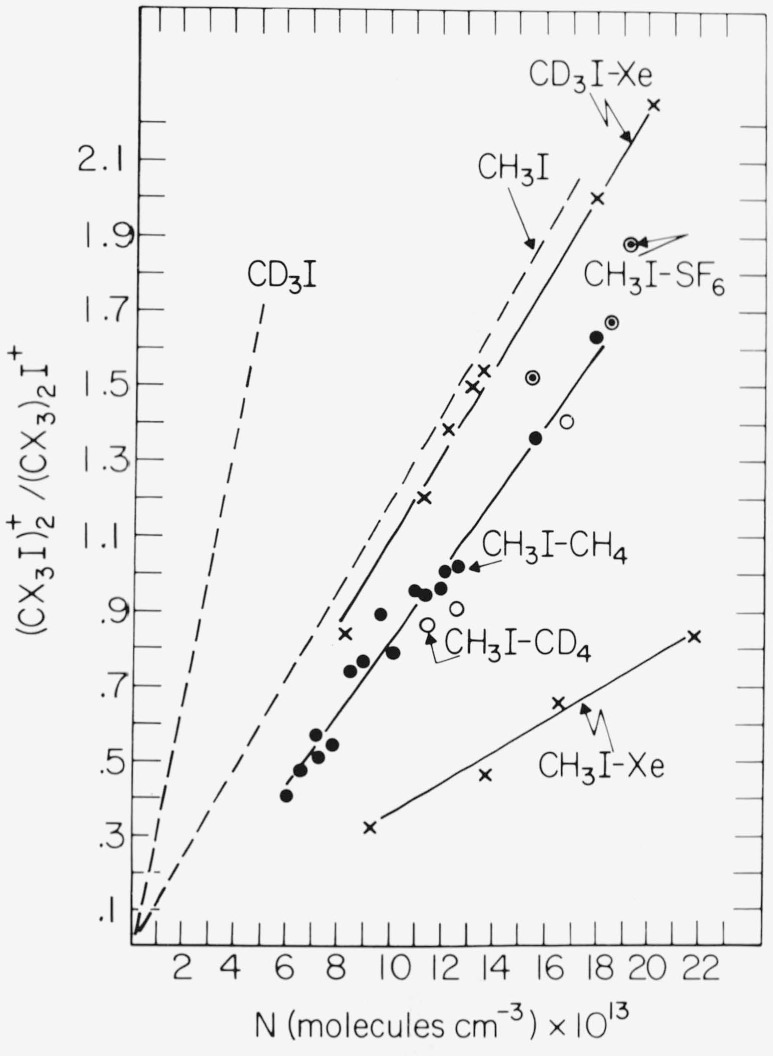
${\left(CX_{3}I\right)}_{2}^{+}/{\left({\text{CX}}_{3}\right)}_{2}I^{+}$ ratio (*X* = *H* or *D*) versus concentration of added *CH*_4_, *CD*_4_, *Xe*, and *SF*_6_.

A number of measurements were also taken to estimate the stabilization efficiencies for other third bodies relative to methyl iodide itself. These results, which were obtained in mixtures containing ~5 percent CH_3_I or CH_3_I in the bulk additive, are shown in figure 4. Approximate relative values in CH_3_I were derived from the additive concentration at which the intensity of ${\left({\text{CH}}_{3}I\right)}_{2}^{+}$ was equal to that for (CH_3_)_2_I^+^. Taking 1.0 as the efficiency for CH_3_I as *M* in the overall reaction

$${\text{CX}}_{3}I^{+}+{\text{CX}}_{3}I\to {\left({\text{CX}}_{3}I\right)}_{2}^{+}\underline{M}{\left({\text{CH}}_{3}I\right)}_{2}^{+}+M$$ (5)

(X = H or D) relative experimental values of 0.68 ± 0.1, 0.65 ± 0.1, 0.31 ± 0.03, 0.87 ± 0.13 were obtained for CH_4_, CD_4_, Xe, and SF_6_ respectively. Mixtures of Xe with CD_3_I gave an efficiency for Xe of 0.30 ± 0.03 relative to CD_3_I = 1.0 in pure CD_3_I. The experimentally determined efficiencies were then modified to take into account the differences in collision frequencies between ${\left({\text{CX}}_{3}I\right)}_{2}^{+*}$ and CX_3_I, CH_4_, etc., which can be calculated directly from Langevin [10] or ADO theory [11]. After applying the appropriate corrections, the relative third-body efficiencies were found to be CH_3_ = 1.0, CH_4_ = 0.57 ± 0.1, CD_4_ = 0.55 ± 0.1, Xe = 0.52 ± 0.05, and SF_6_ = 1.45 ± 0.22 in CH_3_I. Again, taking CD_3_I = 1.0, the relative value for Xe in CD_3_I was determined to be 0.51 ± 0.05, which is equivalent to that derived for Xe in CH_3_I.

### 3.4. Ethyl Iodides

Measurements in C_2_H_5_I, CH_3_CD_2_I, CD_3_CH_2_I, and C_2_D_5_I were taken at 295 K only. The rate coefficients for production of diethyliodium ions, (C_2_X_5_)_2_I^+^, were determined to be 2.0 ± 0.15 × 10^−11^ for all analogues. Stabilization rates (process 4) were again found to increase with increasing deuteration in the order C_2_H_5_I = 1.0, CH_3_CD_2_I = 1.28, CD_3_CH_2_I = 1.79, and C_2_D_5_I = 2.44. On an absolute scale, the third order coefficient for stabilization of ${\left(C_{2}H_{5}I\right)}_{2}^{+*}$ was determined to be 4.1 × 10^−24^, or a factor of 12 higher than that observed in CH_3_I (see table 1).

**Figure 5:**
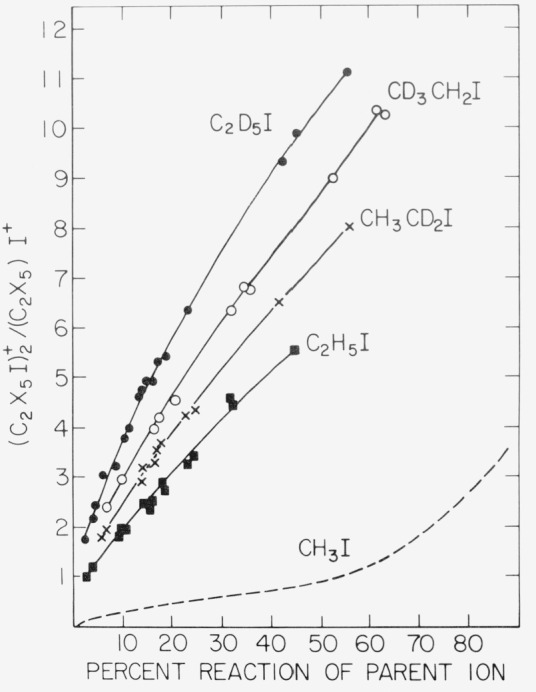
${\left(C_{2}X_{5}I\right)}_{2}^{+}/{\left(C_{2}X_{5}\right)}_{2}I+$ ratio (*X* = *H* or *D*) as a function of the extent reaction of *C*_2_*H*_5_*I*, *CH*_3_*CD*_2_*I*, *CD*_3_*CH*_2_*I*, and *C*_2_*D*_5_*I*.

Biomolecular formation of long-lived dimeric ions was also directly detected in the ethyl iodides. Figure 5, which gives plots of ${\left(C_{2}X_{5}I\right)}_{2}^{+}/{\left(C_{2}X_{5}\right)}_{2}I^{+}$ versus concentration, indicates non-zero intercepts for all of the analogues, including C_2_H_5_I. Observation of these entities corresponds to lifetimes for detected ${\left(C_{2}X_{5}I\right)}_{2}^{+*}$ on the order of several milliseconds, which is the total transit time in our apparatus for ions of these approximate masses. The plots also exhibit downward curvature at higher conversions (higher densities), which is ascribed to formation of undetected trimeric ions having masses beyond the operational range of the quadrupole mass filter used in these experiments (~*m/e* 400). Association ions such as [(C_2_H_5_)_2_I·C2H_5_I]^+^, etc., which correspond to solvation of dialkyliodium ions, were not detected in either the ethyl or methyl iodides at pressures up to 0.1 torr, which was the maximum in the present study.

## 4. Discussion

As expected [12] for association reactions, a negative temperature coefficient was observed for the third order stabilization of ${\left({\text{CX}}_{3}I\right)}_{2}^{+}$ in CH_3_I and CD_3_I (see table 1). However, the variation with temperature does not yield a straight line when *k*(stab.) is plotted versus *T*^−n^ for any values of *n* from 0.1 to 5, although unique fits of this form have been obtained for other systems. The lack of a unique functional form or profound temperature effect in methyl iodide is ascribed to the fact that the stabilization coefficients are extremely high over the range studied, so that no substantial dependence would be anticipated except at much higher temperatures (other studies [12] have been restricted to association reactions having 300 K stabilization coefficients 2–5 orders of magnitude slower than those characteristic of the alkyl iodides). In addition, the ratios of the third order coefficients in CD_3_I and CH_3_I at any temperature are constant at 2.8, which would be expected since the physical factors involved in the stabilization mechanism are presumably the same for both analogues.

Consideration of the relative stabilization efficiencies for the labelled ethyl iodides (table 1) indicates that deuteration on either the methyl or methylene carbon facilitates stabilization (comparison of CH_3_CD_2_I and CD_3_CH_2_I with C_2_H_5_I or C_2_D_5_I). Based on the relative values, attempts were made to calculate the effect of D substitution on both carbons choosing various trial isotope effects at each site. However, no unique self-consistent set of relative or individual contributions compatible with the experimental ratios could be computer-generated due to the fact that all models predicted a substantially higher relative value for CH_3_CD_2_I (≥15 percent higher, which was considered outside the possible error in measurement). The only common result of all calculations was that substitution of D for H on the methylene carbon has a slightly lower effect (~10 percent) than exchanging a D for an H on the methyl group.

Among all of the active vibrational fundamentals in the alkyl halides, the methyl deformation and the methylene rocking modes (ethyl iodide), which lie substantially lower in energy than the C—H stretch, are the most easily excited. The effect of deuteration is to reduce the C—H stretching, CH_3_ deformation, and CH_2_ rocking frequencies by a factor of ~0.3, while the C—I stretching frequency remains essentially unaffected [13]. The large differences observed in the third order stabilization coefficients for the variously labeled iodides are therefore ascribed to a depression of the deformation and rocking frequencies (shift to lower energies and higher state densities) upon deuterium substitution. This depression is manifested by an increased dissociative lifetime for the unstable initial ion-molecule collision complex, as evidenced by the fact that the relative yields of the bimolecularly produced ethyl iodide dimer ions which survived ion transit (extrapolated zero conversion intercepts in figure 4) also appear to increase with increasing deuteration. The lack of isotopic scrambling also indicates that both reacting partners retain their structural integrity within the excited collision complex, facilitating a favorable energy level match between the third body and the excited dimeric ion in the pure halide systems. This favorable condition would account for the magnitude of the stabilization coefficients, which are the highest recorded for third order association reactions at 295 K.

With respect to the measured stabilization efficiencies of other third bodies relative to methyl iodide itself, it is somewhat surprising that methane and Xe exhibit the same relative value (~0.5) since Xe can only deactivate via vibrational to translational energy conversion. Also, CH_4_ and CD_4_ are found to be equally effective even though there is a 30 percent decrease in the vibrational frequencies for CD_4_ relative to CH_4_, which should increase the probability for energy transfer. The low overall efficiency found for methane may be due either to the fact that the duration of the collision between excited dimeric ions and methane is less than the duration with Xe, SF_6_, or CH_3_I (based on relative velocity considerations), or that the active vibrational frequencies in the excited dimers are substantially lower than those associated with the methane fundamentals (≥ ~1000 cm^−1^). Sulfur hexafluoride has both a high mass (146 a.m.u.) and several very low-lying vibrational modes (<800 cm^−1^) which accounts for the observation that it is slightly more effective than CH_3_I itself as a deactivator for ${\left({\text{CH}}_{3}I\right)}_{2}^{+}$.

In conclusion we would like to emphasize the intriguing fact that the stabilization efficiencies for CD_3_I, C_2_D_5_I, and C_6_D_6_ (see table 1) are all 2.6 ± 0.2 times higher than those for the perprotonated analogues. Whether this equivalence is fortuitous, is due to common physical property of the excited dimer cation in these particular systems, or is generally characteristic of polyatomic organic ion-molecule collision complexes which have no highly exothermic or favorable fragmentation channels, deserves further study.

## Figures and Tables

**Table 1:** Rate coefficients for second and third order reactions in methyl and ethyl iodide

System	Temp. (K)	*k*(R)_2_I^+^^a^	*k*(RI)_2_^+^^b^	Relative *k*(RI)_2_^+^^c^
CH_3_I	295 ± 1	2.37 ± 0.12 × 10^−11^	0.33 × 10^−24^	1.0
CH_3_I	220 ± 3	2.46 ± 0.25 × 10^−11^	0.45 × 10^−24^	1.0
CH_3_I	320 ± 1	2.37 ± 0.05 × 10^−11^	0.24 × 10^−24^	1.0
CD_3_I	295 ± 1	2.23 ± 0.05 × 10^−11^	0.94 × 10^−24^	2.8
CD_3_I	220 ± 3	2.04 ± 0.12 × 10^−11^	1.26 × 10^−24^	2.8
CD_3_I	320 ± 1	2.16 ± 0.12 × 10^−11^	0.67 × 10^−24^	2.8
CH_3_I-CD_3_I (1:1)	295 ± 1	2.3 ±0.1 × 10^−11^	0.61 × 10^−24^	1.8
C_2_H_5_I	295 ± 1	2.0 ±0.1 × 10^−11^	4.1 × 10^−24^	1.0
CH_3_CD_2_I	295 ± 1	2.0 ±0.1 × 10^−11^	5.3 × 10^−24^	1.28
CD_3_CH_2_I	295 ± 1	2.0 ±0.1 × 10^−11^	7.4 × 10^−24^	1.79
C_2_D_5_I	295 ± 1	2.0 ±0.1 × 10^−11^	10.1 × 10^−24^	2.44
C_6_H_6_	295 ± 1		0.194 × 10^−24^^d^	1.0
C_6_D_6_	295 ± 1		0.526 × 10^−24^^d^	2.71

a Units are cm^3^ molecule ^−1^ s^−1^.

b Units are cm^6^ molecule^−2^ s^−1^.

c Relative value taking *k* = 1.0 for unlabeled analogue at that temperature.

d Values taken from Reference 5.

## Footnotes

1 Figures in brackets indicate the literature references at the end of this paper.

2 1 torr = 133.32 Nm^−2^.

3 Bimolecular units are cm^3^ molecule^−1^ s^−1^.
